# Genome Sequence of *Microbacterium* sp. Strain TPU 3598, a Lumichrome Producer

**DOI:** 10.1128/genomeA.00204-17

**Published:** 2017-04-20

**Authors:** Kazunori Yamamoto, Yasuhisa Asano

**Affiliations:** a Biotechnology Research Center and Department of Biotechnology, Toyama Prefectural University, Kurokawa, Imizu, Toyama, Japan; b Asano Active Enzyme Molecule Project, ERATO, JST, Kurokawa, Imizu, Toyama, Japan

## Abstract

We report here the genome sequence of *Microbacterium* sp. strain TPU 3598, previously described as a producer of lumichrome. The sequenced genome size is 3,787,270 bp, the average G+C content is 68.39%, and 3,674 protein-coding sequences are predicted.

## GENOME ANNOUNCEMENT

*Microbacterium* sp. strain TPU 3598 was isolated from soil as an efficient producer of lumichrome from riboflavin in our previous study (1). Lumichrome is available as a photosensitizer (2, 3) and fluorescent dye (4, 5). The strain exhibited high production of lumichrome by incubation with a medium containing riboflavin, suggesting that the enzyme catalyzing the conversion of riboflavin to lumichrome was inducibly produced. We hypothesized that the lumichrome produced by our strain might be catalyzed by the riboflavin hydrolase, which has been suggested to catalyze the conversion of riboflavin to lumichrome (6). Recently, Xu et al. identified the riboflavin hydrolase RcaE in the riboflavin catabolic pathway of *Microbacterium maritypicum* strain G10 and expressed the gene in *Escherichia coli* BL21(DE3) (7), although they did not show the gene and amino acid sequences. Similar to *M. maritypicum* strain G10, our strain belongs to the genus *Microbacterium* and produces the enzyme catalyzing the conversion of riboflavin to lumichrome. In contrast to *M. maritypicum*, however, our strain is lipase activity negative and Voges-Proskauer test positive (1, 8). Thus, elucidating the induction mechanism of riboflavin hydrolase in *Microbacterium* sp. strain TPU 3598 may be benefical in increasing the biochemical production of lumichrome.

Genomic DNA from *Microbacterium* sp. strain TPU 3598 was prepared from the cultured cells using NucleoBond AXG 100 (Macherey-Nagel, Germany) and NucleoBond buffer set III (Macherey-Nagel). Genome library preparation and sequence analysis were performed using the PacBio RSII platform (Pacific Biosciences, USA) at Beijing Genomics Institute (BGI, China). The sequence analysis yielded 98,855 reads, totaling 687,856,723 bp, with 180-fold coverage of the genome. The sequence reads were assembled using Celera Assembler version 3 (9) into two high-quality scaffolds (chromosome, 3,787,270 bp; plasmid, 35,712 bp). The G+C contents of the chromosome and plasmid were 68.39% and 64.05%, respectively.

The genome and plasmid genes were predicted using Glimmer 3.0 (10) for protein-coding sequences (CDSs). rRNA and tRNA sequences were predicted using RNAmmer (11) and tRNAscan (12), respectively. The genome of *Microbacterium* sp. strain TPU 3598 contained 3,674 open reading frames, 46 tRNAs, and six rRNAs. The plasmid contained 42 open reading frames. The 3,552 CDSs (96.67% of all genome CDSs) were matched to the known genes using the NCBI NR database, and 212 CDSs were classified as enzymes related to the metabolism of cofactors and vitamins using the Kyoto Encyclopedia of Genes and Genomes (KEGG) (13).

This genome sequence will enable the elucidation of mechanisms underlying efficient lumichrome production by *Microbacterium* sp. strain TPU 3598.

### Accession number(s).

This whole-genome sequence has been deposited in DDBJ/ENA/GenBank under the accession numbers AP017975 (chromosome) and AP017976 (plasmid).
